# Supplementary material for: Loss of Parkinson's disease-associated protein CHCHD2 affects mitochondrial crista structure and destabilizes cytochrome c
Source: Nat Commun. 2017 Jun 7;8:15500. doi: 10.1038/ncomms15500 (PMC5467237; doi:10.1038/ncomms15500)
Supplement: Supplementary Information — Supplementary Figures and Supplementary References [file ncomms15500-s1.pdf]

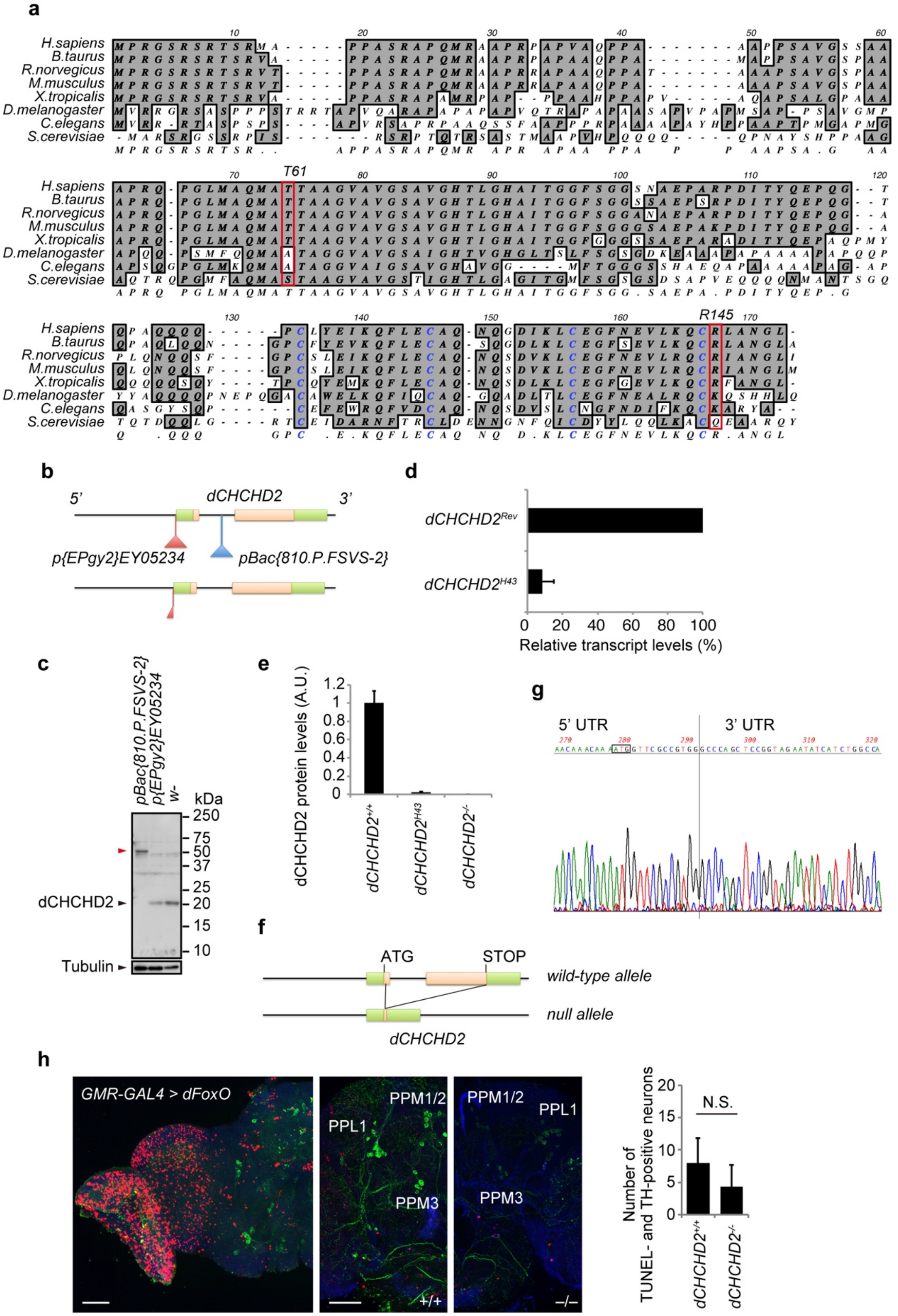
a
H.sapiens
B.taurus
R.norvegicus
M.musculus
X.tropicalis
D.melanogaster
C.elegans
S.cerevisiae
T61
R145
b
5'
dCHCHD2
3'
p{EPgy2}EY05234
pBac{810.P.FSVS-2}
c
pBac{810.P.FSVS-2}
p{EPgy2}EY05234
w-
kDa
250
75
50
37
25
20
15
10
dCHCHD2
Tubulin
d
dCHCHD2^Rev
dCHCHD2^H43
0 20 40 60 80 100
Relative transcript levels (%)
e
dCHCHD2 protein levels (A.U.)
1.2
1
0.8
0.6
0.4
0.2
0
dCHCHD2+/+
dCHCHD2H43
dCHCHD2-/-
f
ATG
STOP
wild-type allele
null allele
dCHCHD2
g
5' UTR
3' UTR
AACAAACAAATGGTTCGCCGTGGGCCCAGCTCCGGTAGAATATCATCTGGCCA
h
GMR-GAL4 > dFoxO
PPL1
PPM1/2
PPM3
+/+
–/–
Number of TUNEL- and TH-positive neurons
20
15
10
5
0
N.S.
dCHCHD2+/+
dCHCHD2-/-

Supplementary Fig. 1. Targeting of the *dCHCHD2* gene.
(**a**) Alignments of full-length CHCHD2 orthologs among species. Grey boxes indicate highly and mildly conserved amino acid residues, and red boxes indicate residues affected in PD. The Cys residues of the twin Cx_9C motif are highlighted in blue. (**b**) The *dCHCHD2* gene locus and transposons (triangles) inserted in *dCHCHD2* (upper). The $dCHCHD2^{H43}$ allele was generated by imprecise excision of *p{EPgy2}EY05234*, and therefore, a 401 bp fragment of *p{EPgy2}EY05234* remains just 1 bp upstream of the transcription start (lower). Green boxes, UTR of *CHCHD2*; Orange boxes, coding regions. (**c**) Expression of dCHCHD2 protein was determined in *w-* and flies harboring *pBac{810.P.FSVS-2}* or *p{EPgy2}EY05234*. Note that the *pBac{810.P.FSVS-2}* transposon with a YFP protein trap, which has a cassette containing YFP with protein tags and splice acceptor and donor sites, is expected to generate a YFP-dCHCHD2 fusion protein because the dCHCHD2 immunosignals were detected at ~ 50 kDa. (**d**) *dCHCHD2* transcript levels in the thorax muscles of dCHCHD2 *Rev* and *H43* flies were estimated by reverse transcription and subsequent quantitative PCR. Relative transcript levels normalized to the housekeeping *RP49* from three independent samples are graphed (mean ± s.e.m.). *H43* reduced expression by approximately 8.5% of Rev. n = 3 from three independent samples. (**e**) dCHCHD2 protein levels in the thorax muscles of dCHCHD2 WT and mutant flies. Relative levels of dCHCHD2 protein normalized to anti-complex V signals from three independent samples are graphed (mean ± s.e.m.). A representative blot image is also shown in Fig. 1b. (**f**) Generation of a *dCHCHD2* null allele. Almost all of the coding regions were removed by the CRISPR/Cas9 technology. (**g**) The deletion region and the junction site (shown by a vertical line) were confirmed by sequencing. A 50-aa polypeptide with a disrupted mitochondrial targeting sequence might be potentially translated from the original translation initiation site of dCHCHD2 (shown in a box), although we could not detect any protein signals in western blot analysis with our anti-dCHCHD2 antibody. (**h**) TUNEL assay of brain samples. (Left) *GMR-GAL4>UAS-dFoxO* crosses at an early pupal stage were used as a positive control. Extensive TUNEL signals were observed in the optic lobes. (Middle and right) In the same experimental conditions, low amounts of TUNEL signal were detected in the whole brains of 45-day-old flies, and there was no difference between $dCHCHD2^{+/+}$ and $dCHCHD2^{-/-}$ (n = 6, p = 0.254 by two-tailed t-test). Representative posterior brain images including TH neuron clusters (PPM1/2, PPM3 and PPL1) are shown. The number of TUNEL-positive TH neurons on both anterior and posterior sides was graphed. Scale bars = 50 μm.

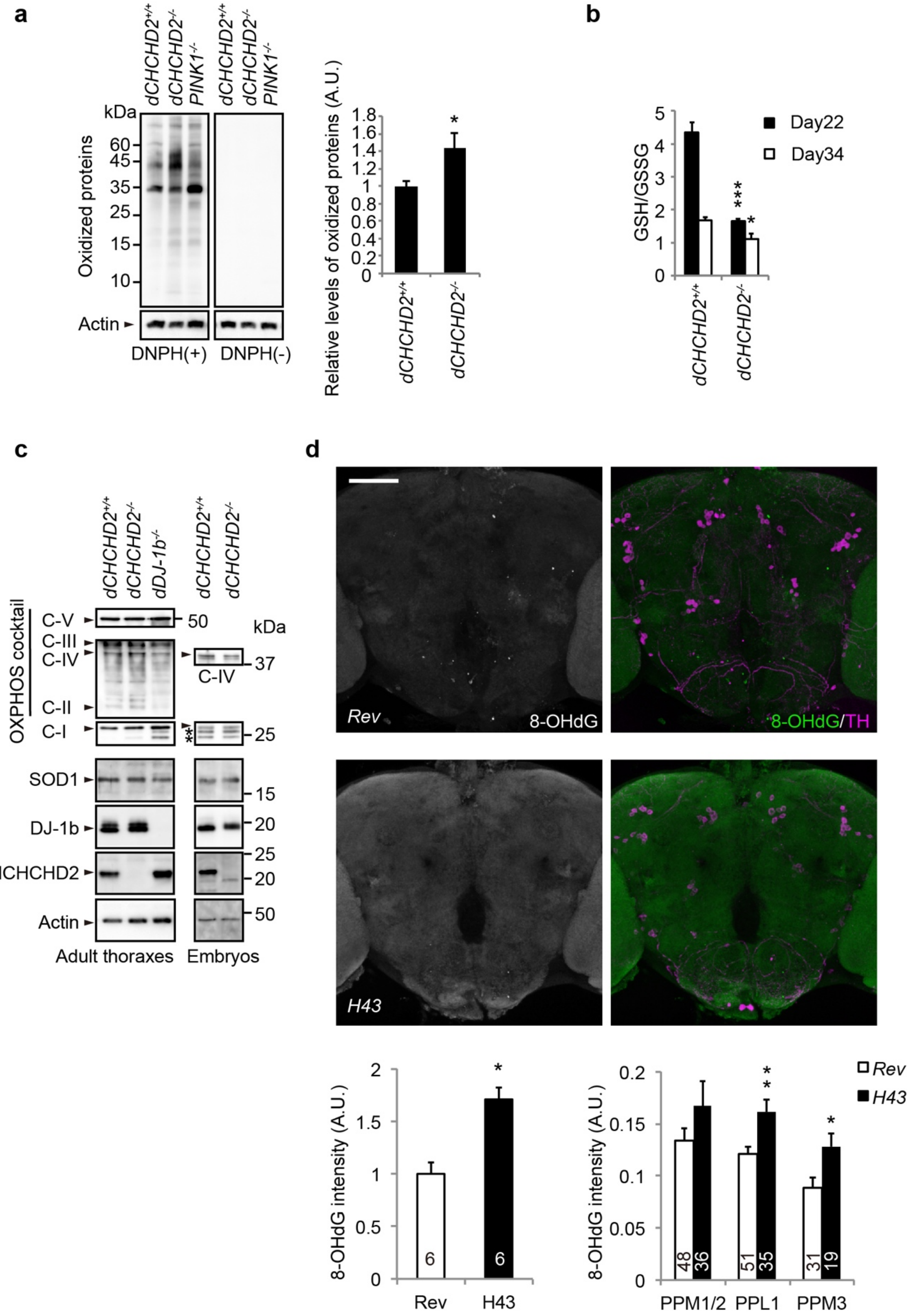
a
dCHCHD2+/+
dCHCHD2-/-
PINK1-/-
kDa
60
45
35
25
15
10
Oxidized proteins
Actin
DNPH(+)
DNPH(-)
Relative levels of oxidized proteins (A.U.)
b
GSH/GSSG
Day22
Day34
c
OXPHOS cocktail
C-V
C-III
C-IV
C-II
C-I
SOD1
DJ-1b
dCHCHD2
Actin
dDJ-1b-/-
Adult thoraxes
Embryos
d
Rev
H43
8-OHdG
8-OHdG/TH
8-OHdG intensity (A.U.)
PPM1/2
PPL1
PPM3

Supplementary Fig. 2. The loss of dCHCHD2 leads to oxidative DNA damage.
(**a**) Detection of protein oxidation by the Oxyblot assay was performed as in Fig. 8e. Thoraxes of 45-day-old flies were used for the Oxyblot assay. Protein oxidation signals were normalized based on Actin, and the signals were increased in *dCHCHD2*-deficient flies (n = 5, **p* = 0.047 by two-tailed *t*-test). Signal specificity was confirmed in blots omitting 2-4-dinitrophenyl hydrazine (DNPH) treatment. (**b**) The GSH/GSSG ratio was reduced in *dCHCHD2*-deficient flies. (n = 4, **p* = 0.020, ****p* = 0.0002 *vs.* age-matched $dCHCHD2^{+/+}$ by two-tailed *t*-test) (**c**) The levels of the OXPHOS proteins, SOD1 and DJ-1b in *dCHCHD2*-deficient flies. Western blot analysis of the thorax tissues of 14-day-old adult flies (left) and embryos (right) used in Fig. 4 and Supplementary Fig. 4 are shown. $DJ\text{-}1b^{-/-}$-deficient flies (*DJ-1b* Δ*93/DJ-1b* Δ*93*) were also used as a control. Note that some of the OXPHOS proteins were not detected in embryos due to less-abundant expression. Asterisks indicate uncharacterized anti-complex I (NDUFS3)-detectable bands. (**d**) Whole brain tissues of 30-day-old flies with the indicated genotypes were stained with anti-8-OHdG and anti-dTH antibodies. Scale bar = 75 µm. Intensity of anti-8-OHdG immunoreactive signals in whole brain (left graph, n = 6, **p* = 0.020 by two-tailed student's *t*-test) and TH-positive regions (right graph, n = 19-51, ***p* = 0.0036, **p* = 0.021 vs. *Rev.* by two-tailed student's *t*-test) were measured and graphed (mean ± s.e.m.). The numbers of samples analyzed are indicated in the graphs.

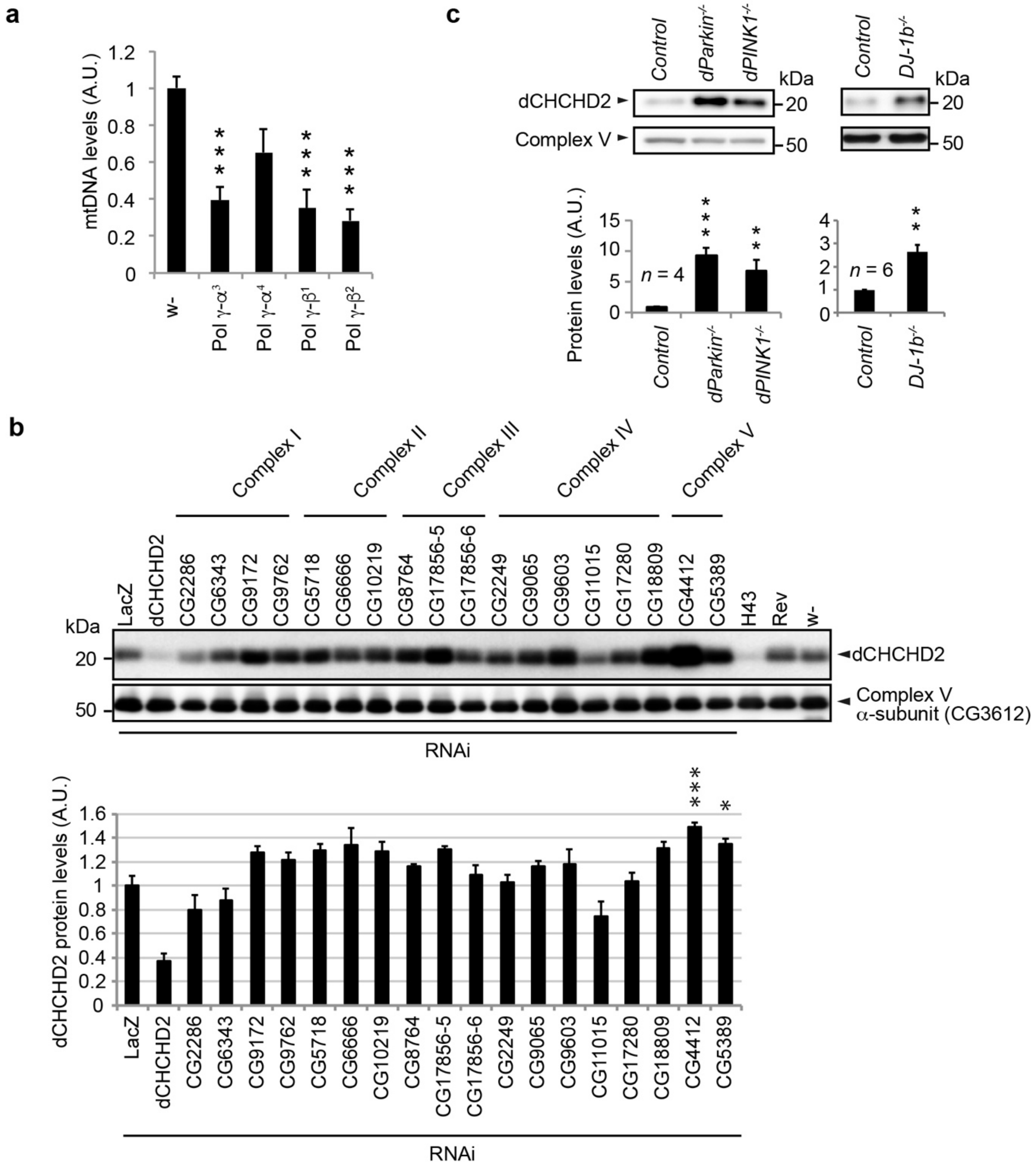

Supplementary Fig. 3. Inactivation of subunits in the mitochondrial respiratory complexes and mitochondria-associated PD genes alters dCHCHD2 expression.
(**a**) Mutations in mitochondrial polymerase γ lead to a loss of mitochondrial DNA (mtDNA). The ratio of mtDNA to genomic DNA of actin was measured by quantitative PCR, and values are normalized to those of the *w-* control (mean ± s.e.m., n = 9 in each group). $***P < 0.0001$

vs. w- (one-way ANOVA with Tukey-Kramer test). (**b**) A series of *UAS*-RNAi fly lines against the respiratory complex subunits, the inhibition of which has been reported to affect lifespan extension and modulation of mitochondrial phenotypes in PD model flies [1, 2, 3, 4, 5], were crossed with the *MHC-GAL4*. Endogenous levels of dCHCHD2 and ATP5a (Complex V, α-subunit) in the thorax muscles were analyzed by western blot. *LacZ* RNAi and *dCHCHD2* RNAi lines served as negative and positive controls, respectively. $dCHCHD2^{H43}$, revertant (Rev), and *w-* were also included as controls. The graph represents relative values (mean ± s.e.m.) of dCHCHD2 signals normalized to ATP5a signals from six independent samples. **p* = 0.041, ****p* = 0.0008 *vs.* LacZ (one-way ANOVA with Tukey-Kramer test). (**c**) The protein levels of dCHCHD2 in *PINK1-*, *parkin-* and *DJ-1b*-deficient flies are analyzed as in (**b**). ***p* = 0.0070 (PINK1), ****p* = 0.0003 (Parkin), ***p* = 0.0011 (DJ-1b) *vs.* Control *w-* by two-tailed student's *t*-test. The graphs represent relative values (mean ± s.e.m., n = 4-6).

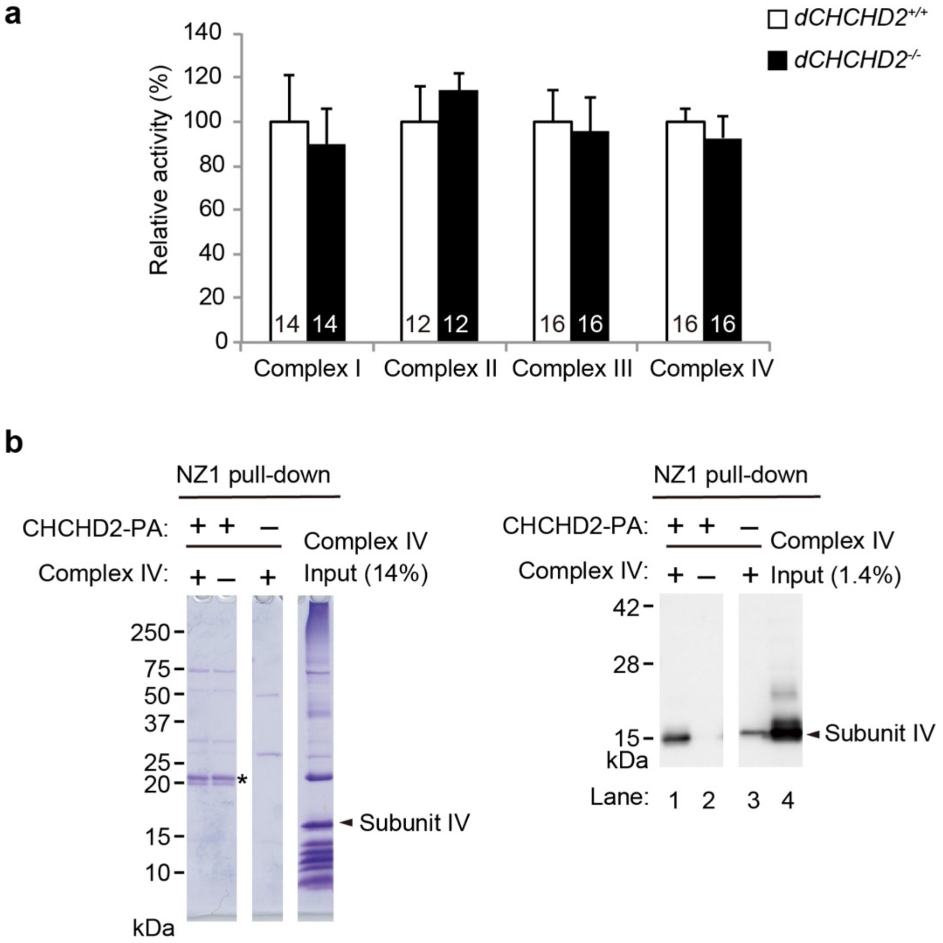

Supplementary Fig. 4. Complex IV regulation is not a primary role of CHCHD2.
(**a**) Measurement of the activities of the four respiratory chain complexes. Mitochondria isolated from thorax muscles were subjected to spectrophotometric analysis of the respiratory chain enzyme activities. All of the activities are presented as percentages of the values of *dCHCHD2*$^{+/+}$ (mean ± s.e.m., n = 12-16 from at least three independent experiments). There were no significant differences in the activity of each complex between the two genotypes (two-tailed *t*-test). The sample size is indicated in the graph. (**b**) CHCHD2 does not significantly bind to complex IV. CHCHD2-PA was purified from cultured cells using anti-PA tag beads (NZ1) and incubated with (Complex IV, +) or without (Complex IV, -) purified complex IV. NZ1 beads alone were also incubated with the same quantity of complex IV in parallel as a mock control. The samples eluted from the beads and input were subjected to SDS-PAGE. Coprecipitated complex IV was evaluated via CBB staining (left) and western blotting with anti-complex IV subunit IV (right). An asterisk indicates bands corresponding to CHCHD2-PA. Western blot analysis indicated that, at most, 0.1% of the input of subunit IV coprecipitated with CHCHD2 (lane 1). However, considering the observation that a similar quantity of subunit IV was detected in the absence of CHCHD2 (lane 3), the binding between CHCHD2 and subunit IV was very weak, if any binding occurred at all.

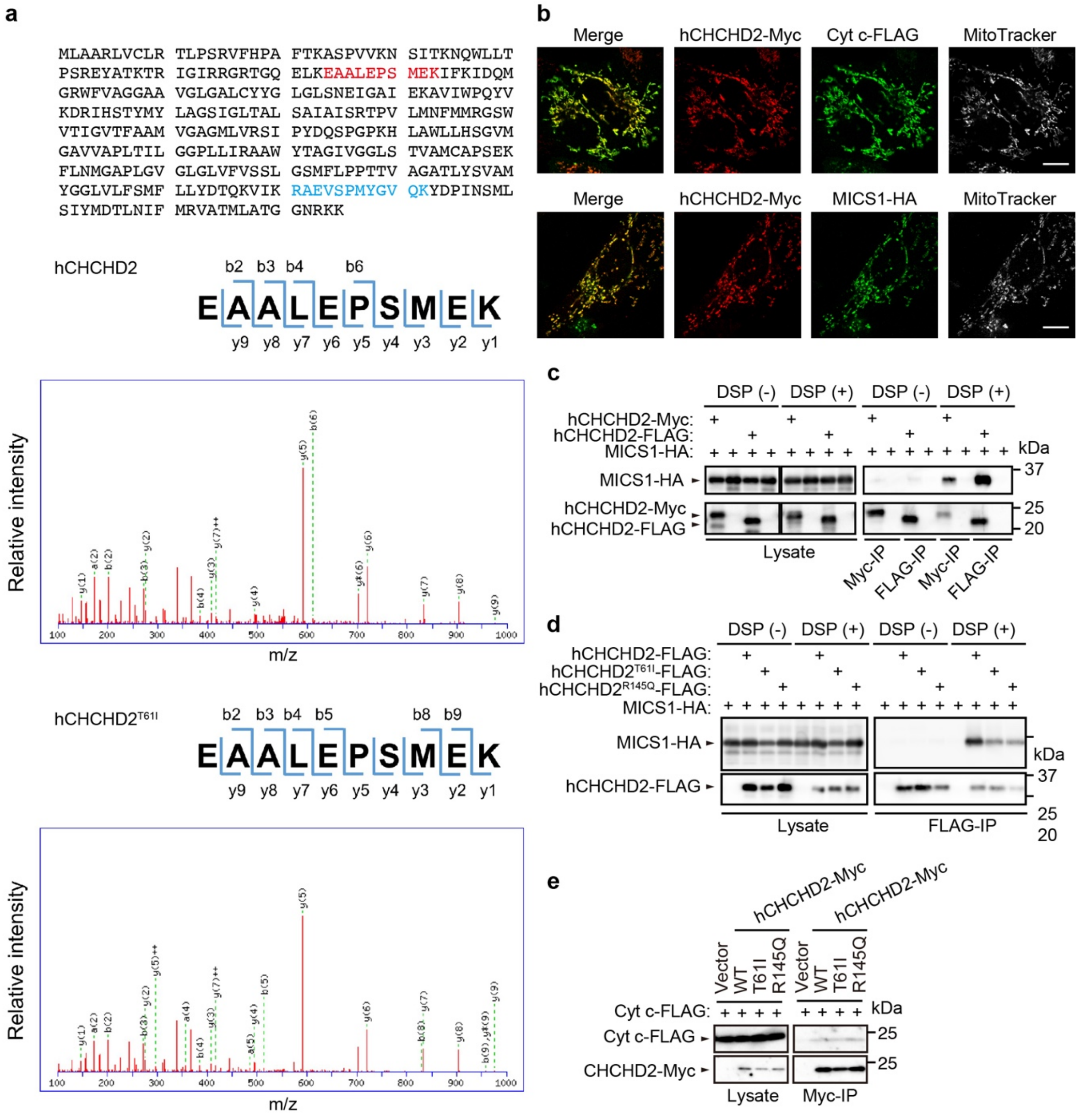

Supplementary Fig. 5. hCHCHD2 interacts with MICS1 in human cultured cells.
(**a**) (Upper) Amino acid sequence of human MICS1. We purified binding proteins using human CHCHD2 WT, T61I and R145Q as probes. Experiments were independently repeated twice, and subsequent mass spectrometry analysis was repeated twice per experiment. A peptide fragment shown in red was detected three and four times in CHCHD2 WT and T61I samples, respectively. A peptide fragment shown in blue was detected once in T61I samples. (Middle and lower) Representative MS/MS spectra assigned to EAALEPSMEK from CHCHD2 WT and T61I samples are shown. (**b**) HeLa cells transfected with hCHCHD2-Myc

in combination with Cyto c-FLAG or with MICS1-HA were co-stained with anti-Myc, anti-FLAG or anti-HA antibodies. Mitochondria were visualized with MitoTracker Deep Red (Thermo Fisher Scientific). Merged images of green and red channels are also shown. Note that the mitochondria were fragmented following MICS1 overexpression, which is consistent with a previous report [6]. Scale bars = 10 μm. (**c**) HEK293T cells were transfected with MICS1-HA with or without hCHCHD2-Myc or hCHCHD2-FLAG. Cells treated with or without 0.5 μg ml^{-1} DSP in PBS for 20 min at RT were lysed and subjected to immunoprecipitation using anti-FLAG or anti-Myc magnetic beads (Medical & Biological Laboratories). MICS1-HA was specifically co-precipitated with hCHCHD2-FLAG or hCHCHD2-Myc. (**d**) HEK293T cells were transfected with MICS1-HA with or without hCHCHD2-FLAG WT or its mutants. Immunoprecipitation using anti-FLAG was performed as in (**c**). The amounts of precipitated R145Q were lower than WT or T61I probably due to its impaired dimerization activity (see also Fig. 7e). (**e**) PD-associated mutations do not affect the binding of CHCHD2 to Cyt c. HEK293T cells were transfected with Cyt c-FALG with or without hCHCHD2-Myc WT or its mutants and treated with 0.5 μg ml^{-1} DSP. Immunoprecipitation using anti-Myc was performed as in (**c**).

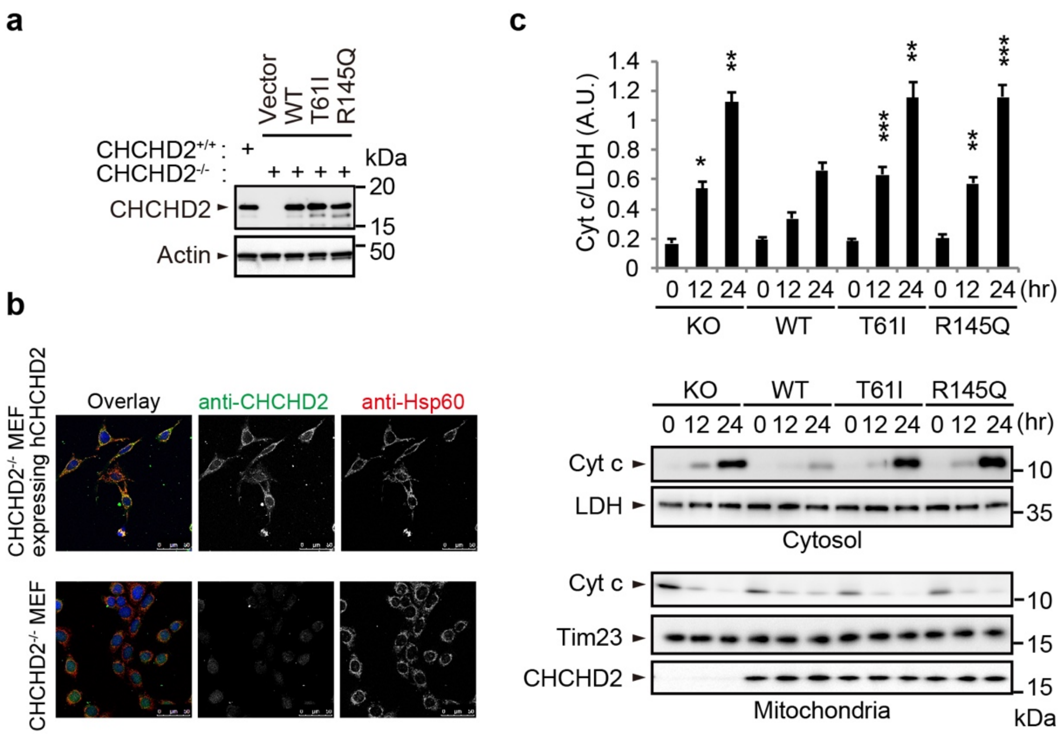

Supplementary Fig. 6. CHCHD2 suppresses Cyt c release upon oxidative stress treatment.

(**a**) MEFs were prepared from *CHCHD2*$^{+/+}$ and *CHCHD2*$^{-/-}$ mouse embryos. An empty vector, hCHCHD2 WT, T61I or R145Q was virally introduced into *CHCHD2*$^{-/-}$ MEFs. CHCHD2 expression levels were comparable in WT and PD mutant cells. (**b**) Human CHCHD2 is localized in mitochondria of *CHCHD2*$^{-/-}$ MEFs. Cells were stained with anti-CHCHD2 and anti-Hsp60 antibodies. Scale bars = 50 µm. (**c**) CHCHD2 suppresses Cyt c release caused by peroxide treatment. *CHCHD2*-deficient MEFs harboring empty vector (KO), hCHCHD2 WT, T61I or R145Q expression plasmid were used, as in Fig. 5c and d. Cells were treated with 50 nM peroxide for the indicated periods of time. The levels of cytosolic Cyt c (mean ± s.e.m.) were determined via western blotting and were normalized to lactate dehydrogenase (LDH) levels. *$p < 0.05$, **$p < 0.01$, ***$p < 0.001$ *vs.* the same periods in WT from seven independent experiments (one-way ANOVA with Tukey-Kramer test).

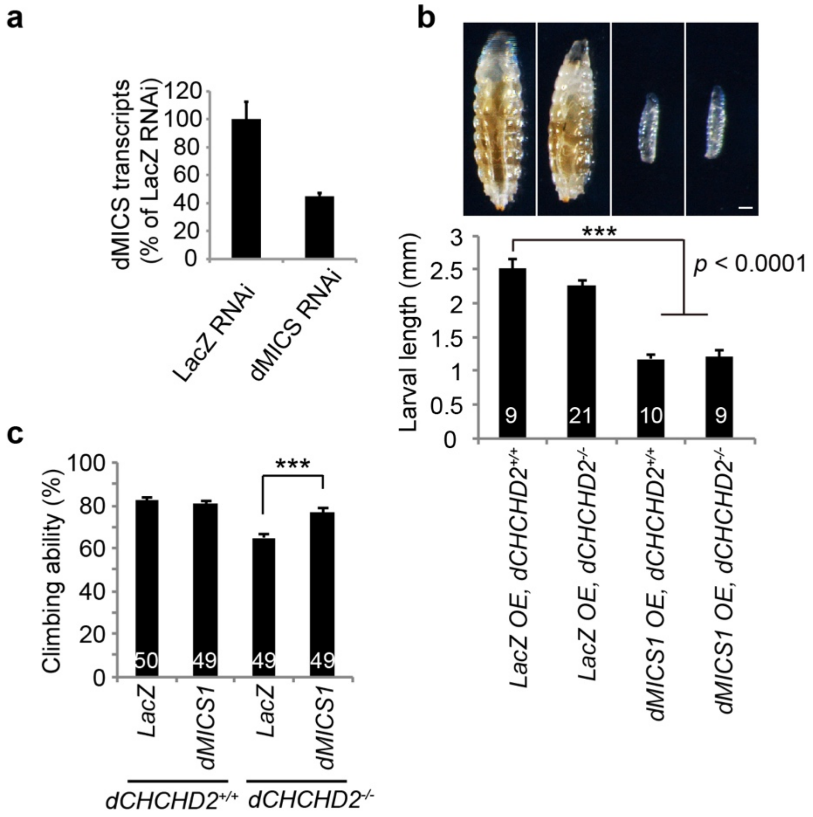

Supplementary Fig. 7. dMICS1 rescues motor defects in *dCHCHD2*$^{-/-}$ flies.
(**a**) Knockdown efficiency of dMICS1. The levels of *dMICS1* transcripts were measured using quantitative RT-PCR, which were then normalized to housekeeping *rp49* levels. Expression of dMICS1 RNAi and LacZ RNAi was induced by the *MHC-GAL4* driver. dMICS1 RNAi reduced the *dMICS1* transcript levels by more than 50% compared with LacZ RNAi. The graph represents relative values (mean ± s.e.m.). n = 3 from three independent samples. (**b**) LacZ or dMICS1 was driven by *Da-GAL4* at 22˚C. The body length of larvae was measured 6 days post hatch. dMICS1 overexpression (OE) led to growth retardation (***$p < 0.0001$ by one-way ANOVA with Tukey-Kramer test) while dCHCHD2 expression did not affect this phenotype and the hatching efficiency. The sample size is indicated in the graph (n = 9-21). Scale bar = 250 µm. (**c**) Climbing defects in *dCHCHD2*$^{-/-}$ flies were rescued by dMICS1 overexpression. dMICS1 was expressed in the muscle tissues using the *MHC-GAL4* driver. Thirty-day-old flies were analyzed (mean ± s.e.m.). Twenty trials with 49-50 flies from three independent experiments. ***$p = 0.0001$.

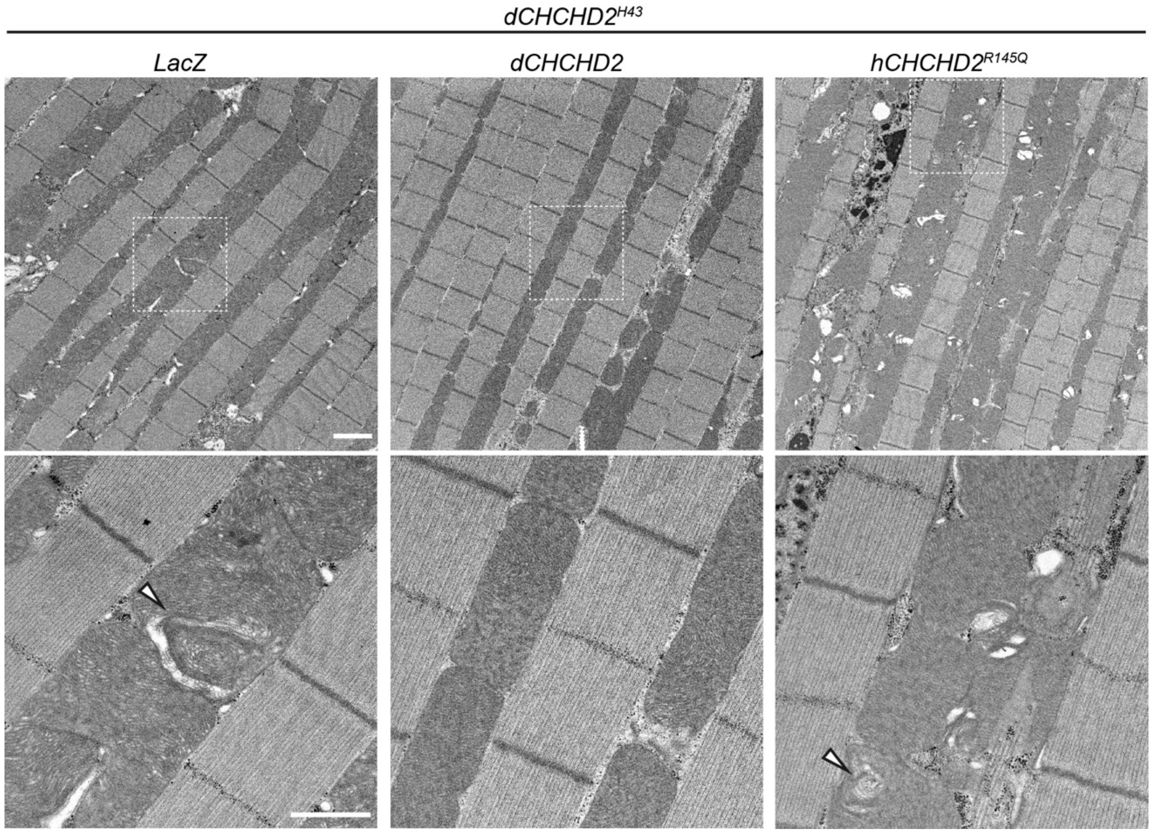

Supplementary Fig. 8. Mitochondrial swirl phenotypes in *dCHCHD2* mutant flies are rescued by dCHCHD2 introduction.

TEM images of the indirect flight muscles of 14-day-old flies with the indicated genotypes. (Lower) Higher magnification images of boxed regions in upper panels. White arrowheads indicate swirling cristae. Scale bars = 2 μm (upper) and 1 μm (lower), respectively.

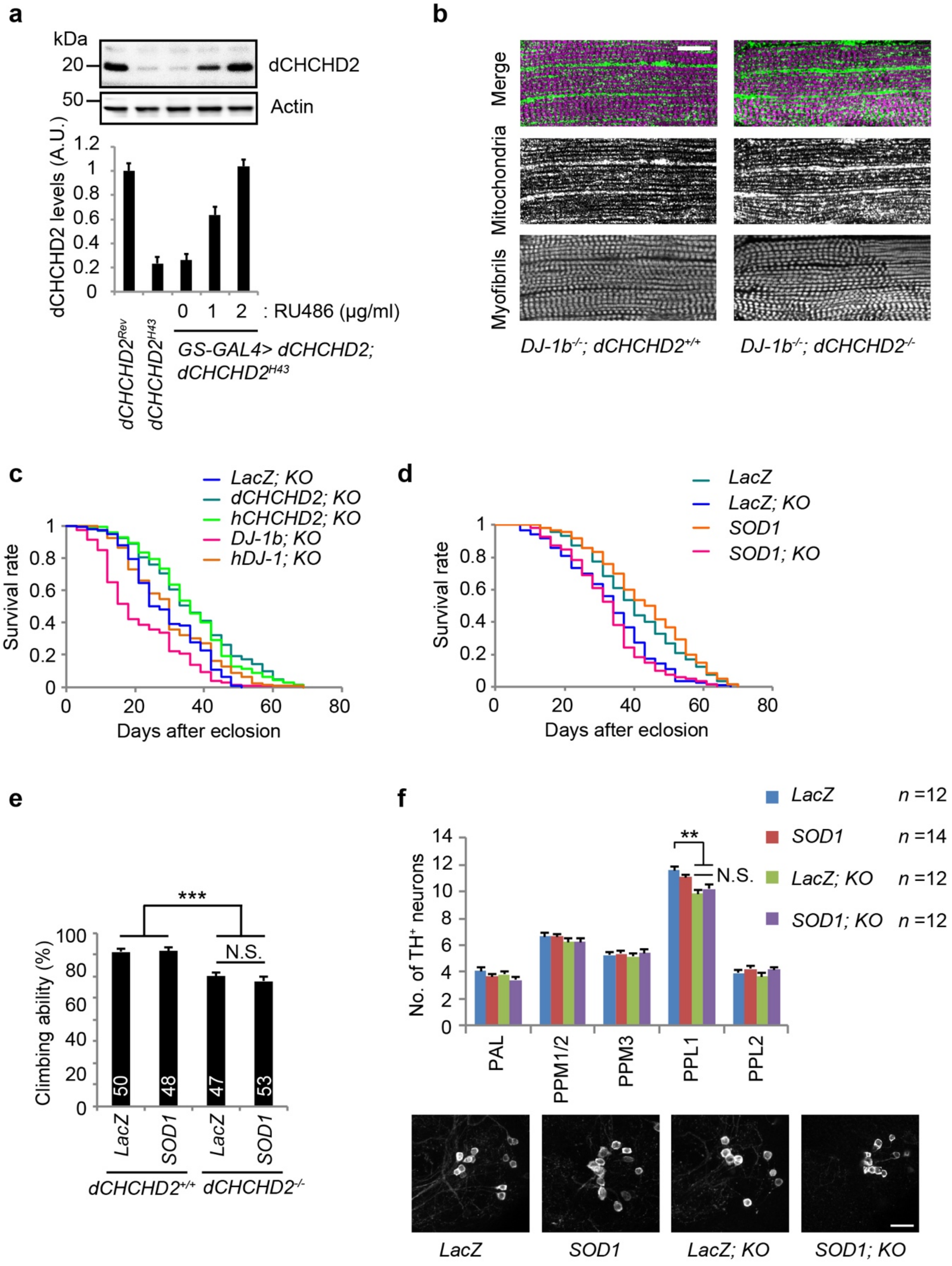
a
kDa
20
50
dCHCHD2
Actin
dCHCHD2 levels (A.U.)
1.2
1
0.8
0.6
0.4
0.2
0
0 1 2 : RU486 (μg/ml)
dCHCHD2Rev
dCHCHD2H43
GS-GAL4> dCHCHD2; dCHCHD2H43
b
Merge
Mitochondria
Myofibrils
DJ-1b-/-; dCHCHD2+/+
DJ-1b-/-; dCHCHD2-/-
c
LacZ; KO
dCHCHD2; KO
hCHCHD2; KO
DJ-1b; KO
hDJ-1; KO
Survival rate
Days after eclosion
d
LacZ
LacZ; KO
SOD1
SOD1; KO
Survival rate
Days after eclosion
e
***
N.S.
Climbing ability (%)
50
48
47
53
LacZ
SOD1
LacZ
SOD1
dCHCHD2+/+
dCHCHD2-/-
f
LacZ n =12
SOD1 n =14
LacZ; KO n =12
SOD1; KO n =12
**
N.S.
No. of TH+ neurons
PAL
PPM1/2
PPM3
PPL1
PPL2
LacZ
SOD1
LacZ; KO
SOD1; KO

Supplementary Fig. 9. DJ-1 and SOD1 do not affect the mitochondrial phenotypes of *dCHCHD2*-deficient flies.

(**a**) Pharmacological induction of dCHCHD2 in *Drosophila*. dCHCHD2 was induced in adult flies crossed with the ubiquitous *daughterless*–Gene-Switch driver (*Da-GS*) by raising flies in media containing the indicated amounts of RU486 (0, 1.0, and 2.0 μg ml^{-1}) for 3 days. Endogenous dCHCHD2 levels (mean ± s.e.m., n = 6) in *dCHCHD2*^{Rev} and *dCHCHD2*^{H43} flies are also shown. Actin served as a loading control. (**b**) Mitochondrial morphology of *DJ-1b*-deficient flies with or without dCHCHD2 activity. Fluorescent images of the indirect flight muscles of 7-day-old adult male flies with the indicated genotypes are shown as in Fig. 9a. Scale bar = 20 µm. The genotypes used were as follows: *dCHCHD2*$^{+/+}$*/Y; MHC-GAL4, UAS-mitoGFP/+*; *DJ-1b* Δ*93/DJ-1b* Δ*93* (*DJ-1b*$^{-/-}$*; dCHCHD2*$^{+/+}$), *dCHCHD2*^{null}*/Y; MHC-GAL4, UAS-mitoGFP/+*; *DJ-1b* Δ*93/DJ-1b* Δ*93* (*DJ-1b*$^{-/-}$*; dCHCHD2*$^{-/-}$). (**c**) DJ-1 overexpression does not extend lifespan of *dCHCHD2*-deficient flies. *hDJ-1; KO vs. LacZ; KO, p* = 0.1805; *DJ-1b; KO vs. LacZ; KO, p* < 0.0001; *dCHCHD2; KO vs. LacZ; KO, p* < 0.0001; *hCHCHD2; KO vs. LacZ; KO, p* < 0.0001 by log-rank test; n = 140 male flies. The genotypes used were as follows: *dCHCHD2*^{null}*/Y; Da-GAL4/UAS-LacZ* (*LacZ; KO*), *dCHCHD2*^{null}*/Y; Da-GAL4/UAS-dCHCHD2* (*dCHCHD2; KO*), *dCHCHD2*^{null}*/Y; Da-GAL4/UAS-hCHCHD2* (*hCHCHD2; KO*), *dCHCHD2*^{null}*/Y; UAS-DJ-1b/+; Da-GAL4/+* (*DJ-1b; KO*), *dCHCHD2*^{null}*/Y; UAS-hDJ-1/+; Da-GAL4/+* (*hDJ-1; KO*). (**d**) SOD1 overexpression does not extend lifespan of *dCHCHD2*-deficient flies (*p* = 0.398, *SOD1; KO vs. LacZ; KO* by log-rank test; n = 120 male flies). (**e**) SOD1 failed to rescue the climbing defects in *dCHCHD2*$^{-/-}$ flies. Forty-five-day-old flies were analyzed (mean ± s.e.m.). ***p < 0.0001; N.S., not significant (one-way ANOVA with Tukey-Kramer test). Twenty trials with 47-53 flies from three independent experiments. (**f**) The number of the indicated cluster of DA neurons (mean ± s.e.m., n = 12-14) from 30-day-old flies was counted and representative images of the PPL1 DA neurons visualized with anti-dTH are shown. Scale bar = 15 μm. **p = 0.004; N.S., not significant (one-way ANOVA with Tukey-Kramer test). The genotypes used in (**d**-**f**) were as follows: *+/Y; Da-GAL4/UAS-LacZ* (*LacZ*), *+/Y; Da-GAL4/UAS-SOD1* (*SOD1*), *dCHCHD2*^{null}*/Y; Da-GAL4/UAS-LacZ* (*LacZ; KO*), *dCHCHD2*^{null}*/Y; Da-GAL4/UAS-SOD1* (*SOD1; KO*).

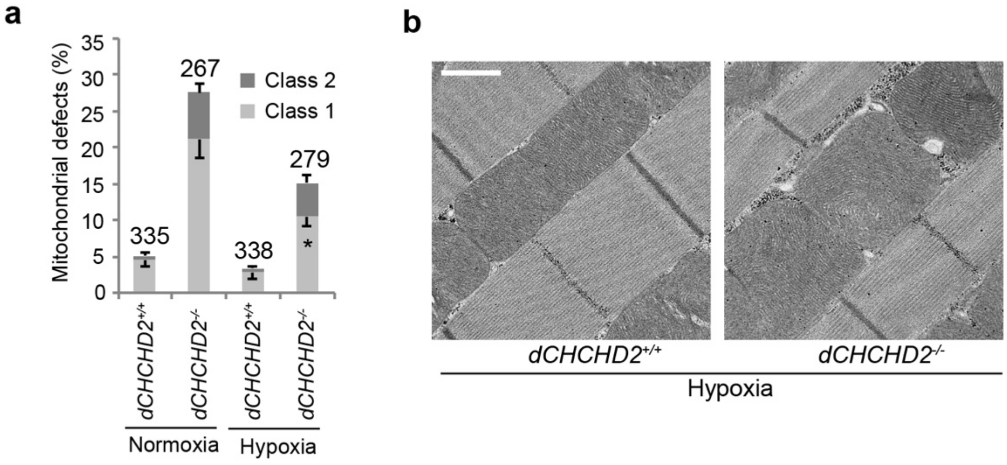

Supplementary Fig. 10. Hypoxia improves the *dCHCHD2* mitochondrial phenotypes. (**a, b**) Newly emerged adult flies were cultured under 3% O_2 (for hypoxia) or 21% O_2 (for normoxia) for 16 days. (**a**) Mitochondria with abnormal cristae or degenerating mitochondria were counted as shown in Fig. 1e (mean ± s.e.m.). n = 267-338 from three independent samples. The sample size is indicated in the graph. *$p = 0.032$ *vs*. normoxia of *dCHCHD2*$^{-/-}$ (one-way ANOVA with Tukey-Kramer test). (**b**) TEM images of the indirect flight muscles of adult flies with the indicated genotypes. Scale bar = 1 μm.

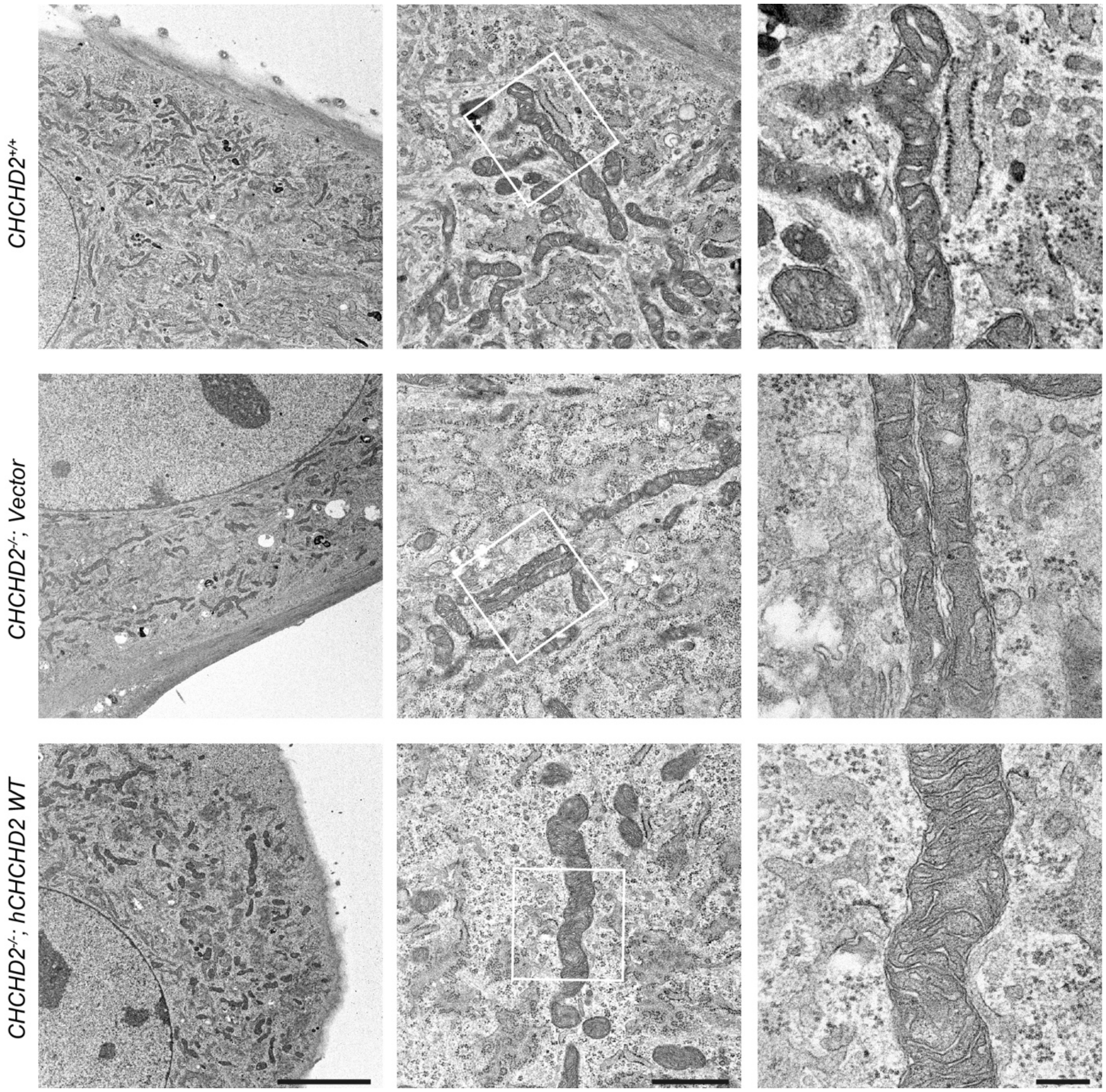

Supplementary Fig. 11. Mitochondrial ultrastructure of *CHCHD2*$^{+/+}$ and *CHCHD2*$^{-/-}$ MEFs.

Representative TEM images of mitochondria in *CHCHD2*$^{+/+}$ MEFs (upper) and *CHCHD2*$^{-/-}$ MEFs retrovirally transfected with pMXs-puro harboring hCHCHD2 (lower) or with pMXs-puro empty vector (middle). (Right) Higher magnification images of boxed regions in center panels. Scale bars = 5 μm (left), 1 μm (center) and 200 nm (right), respectively.

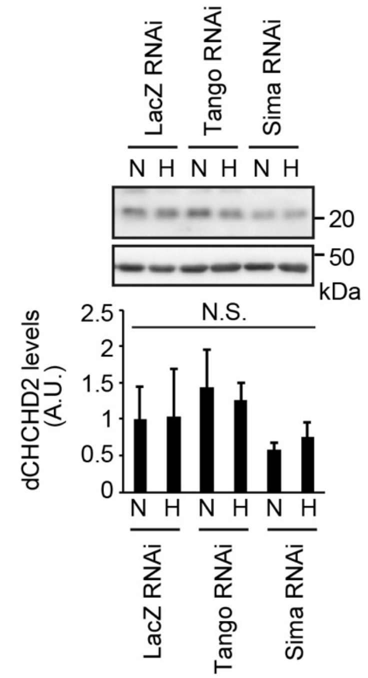

Supplementary Fig. 12. Hypoxia and HIF-1 do not affect dCHCHD2 expression.
(**a**) Newly emerged adult flies were cultured under 3% O_2 (hypoxia, H) or 21% O_2 (normoxia, N) for 5 days. HIF-1α/Sima or HIF-1ß/Tango RNAi was driven by *MHC-GAL4*. Actin served as a loading control. LacZ RNAi was used as a control line. The graph represents relative band intensities of dCHCHD2 normalized to actin (mean ± s.e.m.). n = 3 independent experiments. N.S., not significant (one-way ANOVA with Tukey-Kramer test).

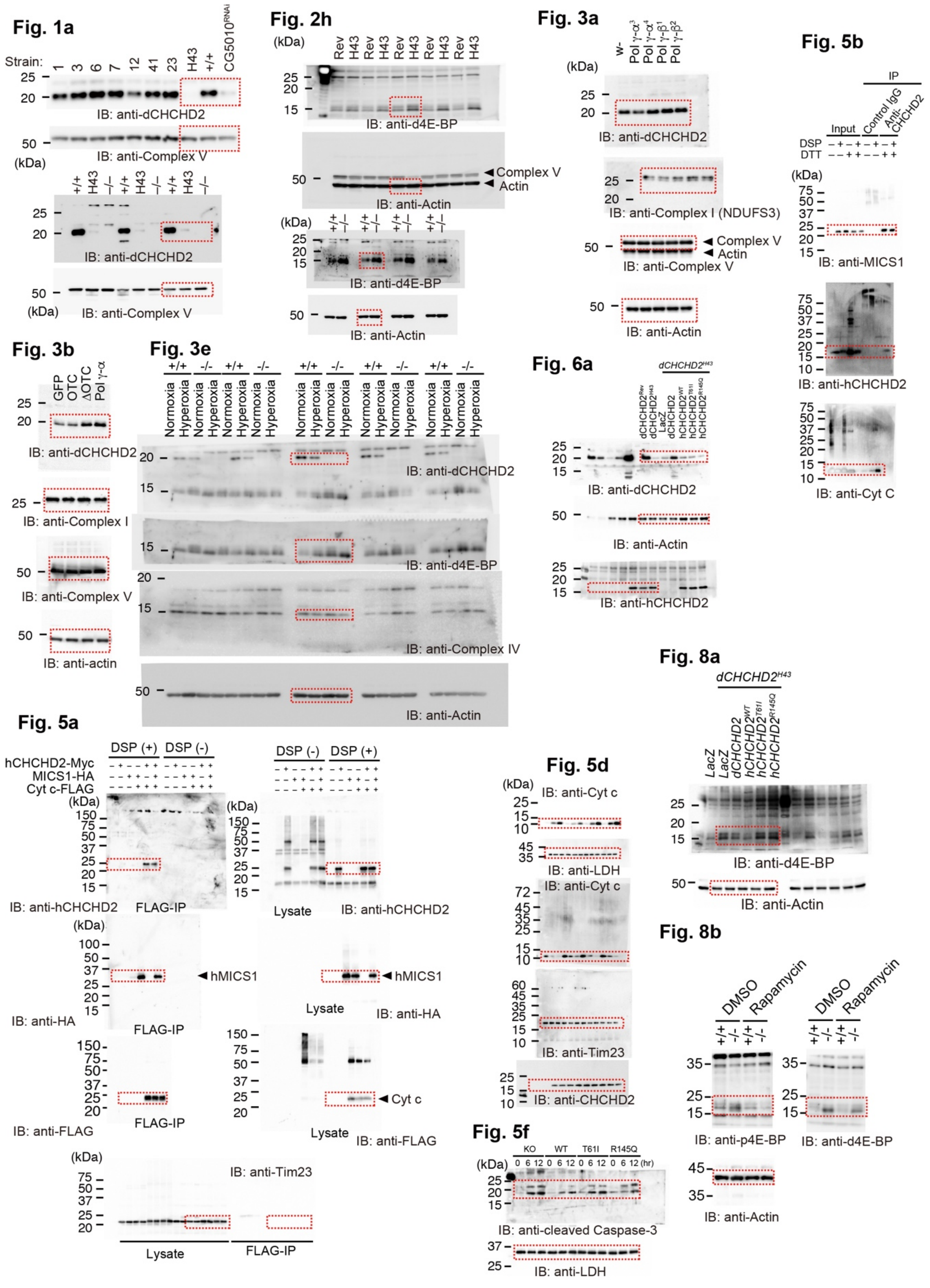
Fig. 1a
Strain: 1 3 6 7 12 41 23 H43 +/+ CG5010RNAi
IB: anti-dCHCHD2
IB: anti-Complex V
(kDa)
+/+ H43 −/− +/+ H43 −/− +/+ H43 −/−
IB: anti-dCHCHD2
IB: anti-Complex V
Fig. 2h
Rev H43 Rev H43 Rev H43 Rev H43 Rev H43
IB: anti-d4E-BP
Complex V
Actin
IB: anti-Actin
IB: anti-d4E-BP
IB: anti-Actin
Fig. 3a
w- Pol γ-α3 Pol γ-α4 Pol γ-β1 Pol γ-β2
IB: anti-dCHCHD2
IB: anti-Complex I (NDUFS3)
Complex V
Actin
IB: anti-Complex V
IB: anti-Actin
Fig. 5b
IP
Input Control IgG Anti-CHCHD2
DSP
DTT
IB: anti-MICS1
IB: anti-hCHCHD2
IB: anti-Cyt C
Fig. 3b
GFP OTC ΔOTC Pol γ-α
IB: anti-dCHCHD2
IB: anti-Complex I
IB: anti-Complex V
IB: anti-actin
Fig. 3e
+/+ -/- +/+ -/- +/+ -/- +/+ -/- +/+ -/-
Normoxia Hyperoxia
IB: anti-dCHCHD2
IB: anti-d4E-BP
IB: anti-Complex IV
IB: anti-Actin
Fig. 6a
dCHCHD2H43
dCHCHD2Rev dCHCHD2H43 LacZ dCHCHD2 hCHCHD2WT hCHCHD2T61I hCHCHD2R145Q
IB: anti-dCHCHD2
IB: anti-Actin
IB: anti-hCHCHD2
Fig. 8a
dCHCHD2H43
LacZ LacZ dCHCHD2 hCHCHD2WT hCHCHD2T61I hCHCHD2R145Q
IB: anti-d4E-BP
IB: anti-Actin
Fig. 5a
DSP (+) DSP (-)
hCHCHD2-Myc
MICS1-HA
Cyt c-FLAG
IB: anti-hCHCHD2 FLAG-IP
DSP (-) DSP (+)
Lysate IB: anti-hCHCHD2
IB: anti-HA FLAG-IP
hMICS1
Lysate IB: anti-HA
IB: anti-FLAG FLAG-IP
Cyt c
Lysate IB: anti-FLAG
IB: anti-Tim23
Lysate FLAG-IP
Fig. 5d
IB: anti-Cyt c
IB: anti-LDH
IB: anti-Cyt c
IB: anti-Tim23
IB: anti-CHCHD2
Fig. 5f
KO WT T61I R145Q
0 6 12 0 6 12 0 6 12 0 6 12 (hr)
IB: anti-cleaved Caspase-3
IB: anti-LDH
Fig. 8b
DMSO Rapamycin DMSO Rapamycin
+/+ -/- +/+ -/-
IB: anti-p4E-BP
IB: anti-d4E-BP
IB: anti-Actin

Supplementary Figure 13. The full-length images of western blot results from the main figures.

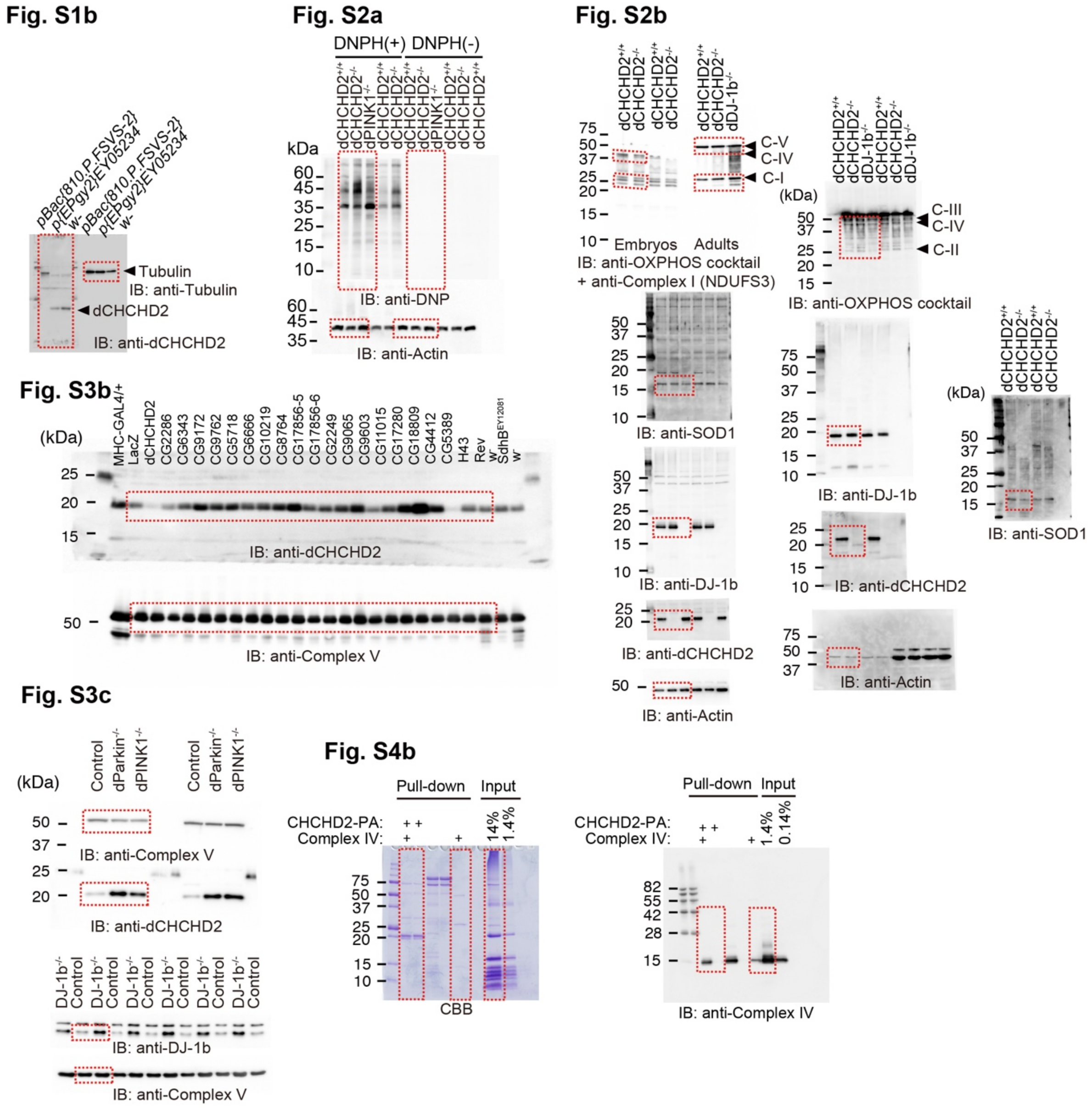

Supplementary Fig. 14. The full-length images of western blot results from the supplementary figures 1-4.

Supplementary Fig 15

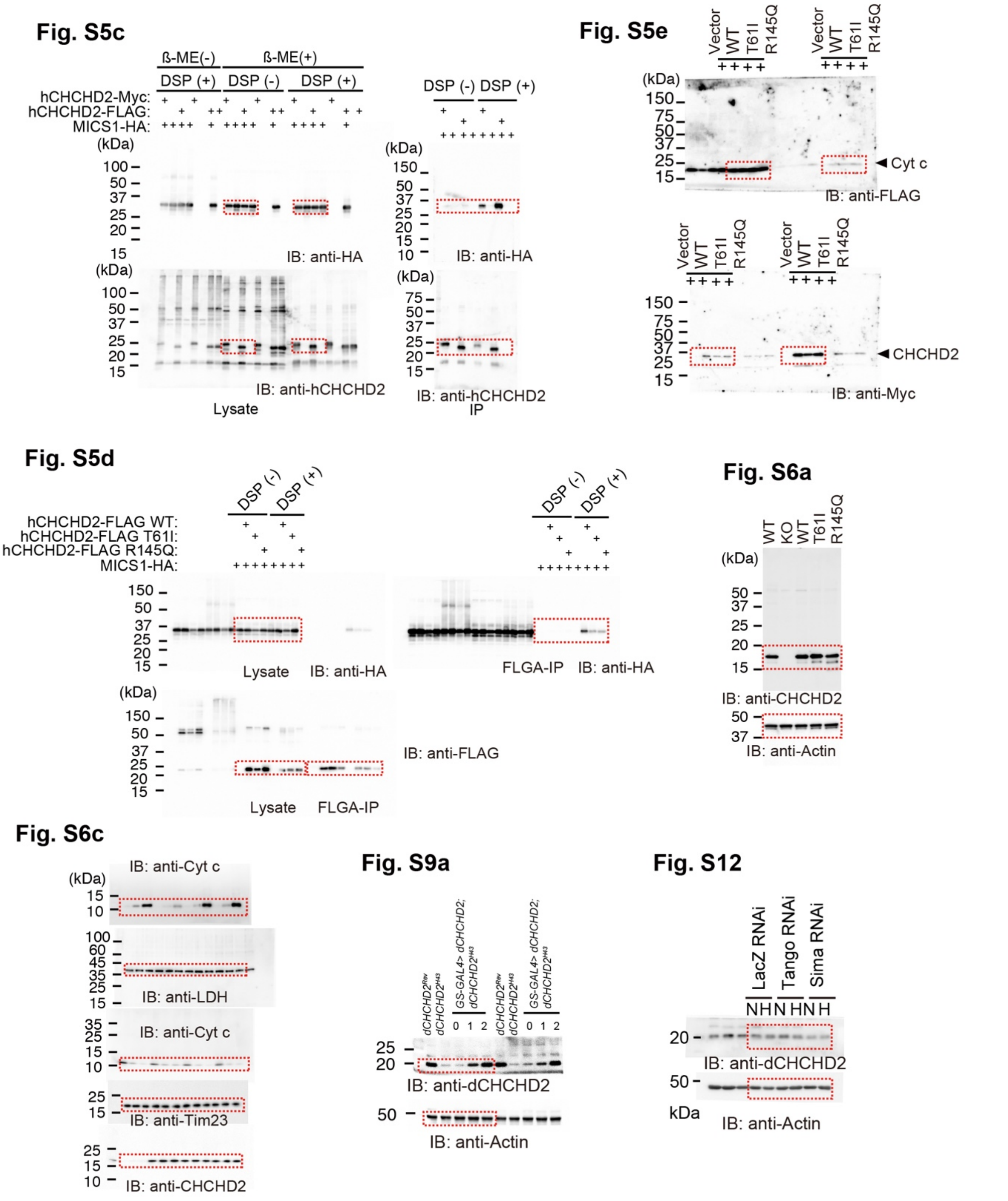

Supplementary Fig. 15. The full-length images of western blot results from the supplementary figures 5-9.

Supplementary References

1. Copeland JM*, et al.* Extension of Drosophila life span by RNAi of the mitochondrial respiratory chain. *Curr. Biol.* **19**, 1591-1598 (2009).

2. Pogson JH*, et al.* The complex I subunit NDUFA10 selectively rescues Drosophila pink1 mutants through a mechanism independent of mitophagy. *PLoS Genet.* **10**, e1004815 (2014).

3. Morais VA*, et al.* PINK1 loss-of-function mutations affect mitochondrial complex I activity via NdufA10 ubiquinone uncoupling. *Science* **344**, 203-207 (2014).

4. Wu Z*, et al.* Tricornered/NDR kinase signaling mediates PINK1-directed mitochondrial quality control and tissue maintenance. *Genes Dev.* **27**, 157-162 (2013).

5. Owusu-Ansah E, Song W, Perrimon N. Muscle mitohormesis promotes longevity via systemic repression of insulin signaling. *Cell* **155**, 699-712 (2013).

6. Oka T*, et al.* Identification of a novel protein MICS1 that is involved in maintenance of mitochondrial morphology and apoptotic release of cytochrome c. *Mol. Biol. Cell* **19**, 2597-2608 (2008).
